# iPSC-derived cells for whole liver bioengineering

**DOI:** 10.3389/fbioe.2024.1338762

**Published:** 2024-02-07

**Authors:** Kayque Alves Telles-Silva, Lara Pacheco, Fernanda Chianca, Sabrina Komatsu, Caroline Chiovatto, Mayana Zatz, Ernesto Goulart

**Affiliations:** ^1^ Human Genome and Stem-Cell Research Center (HUG-CEL), Institute of Biosciences, University of Sao Paulo, Sao Paulo, Brazil; ^2^ Department of Pharmaceutical Chemistry, Small Molecule Discovery Center, Genentech Hall, University of California, San Francisco, San Francisco, CA, United States

**Keywords:** liver, bioengineering, human induced pluripotent stem cells, decellularization, bioprinting

## Abstract

Liver bioengineering stands as a prominent alternative to conventional hepatic transplantation. Through liver decellularization and/or bioprinting, researchers can generate acellular scaffolds to overcome immune rejection, genetic manipulation, and ethical concerns that often accompany traditional transplantation methods, *in vivo* regeneration, and xenotransplantation. Hepatic cell lines derived from induced pluripotent stem cells (iPSCs) can repopulate decellularized and bioprinted scaffolds, producing an increasingly functional organ potentially suitable for autologous use. In this mini-review, we overview recent advancements *in vitro* hepatocyte differentiation protocols, shedding light on their pivotal role in liver recellularization and bioprinting, thereby offering a novel source for hepatic transplantation. Finally, we identify future directions for liver bioengineering research that may allow the implementation of these systems for diverse applications, including drug screening and liver disease modeling.

## Graphical Abstract

**Figure:**
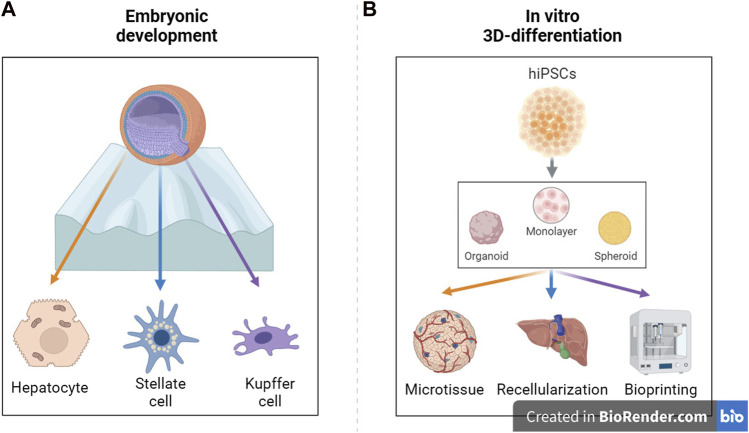
Schematic representation of *in vitro* differentiation protocols **(A)** and bioengineering applications **(B)**. hiPSC = human induced pluripotent stem cell. (Created with BioRender.com.

## Introduction

**FIGURE 1:**
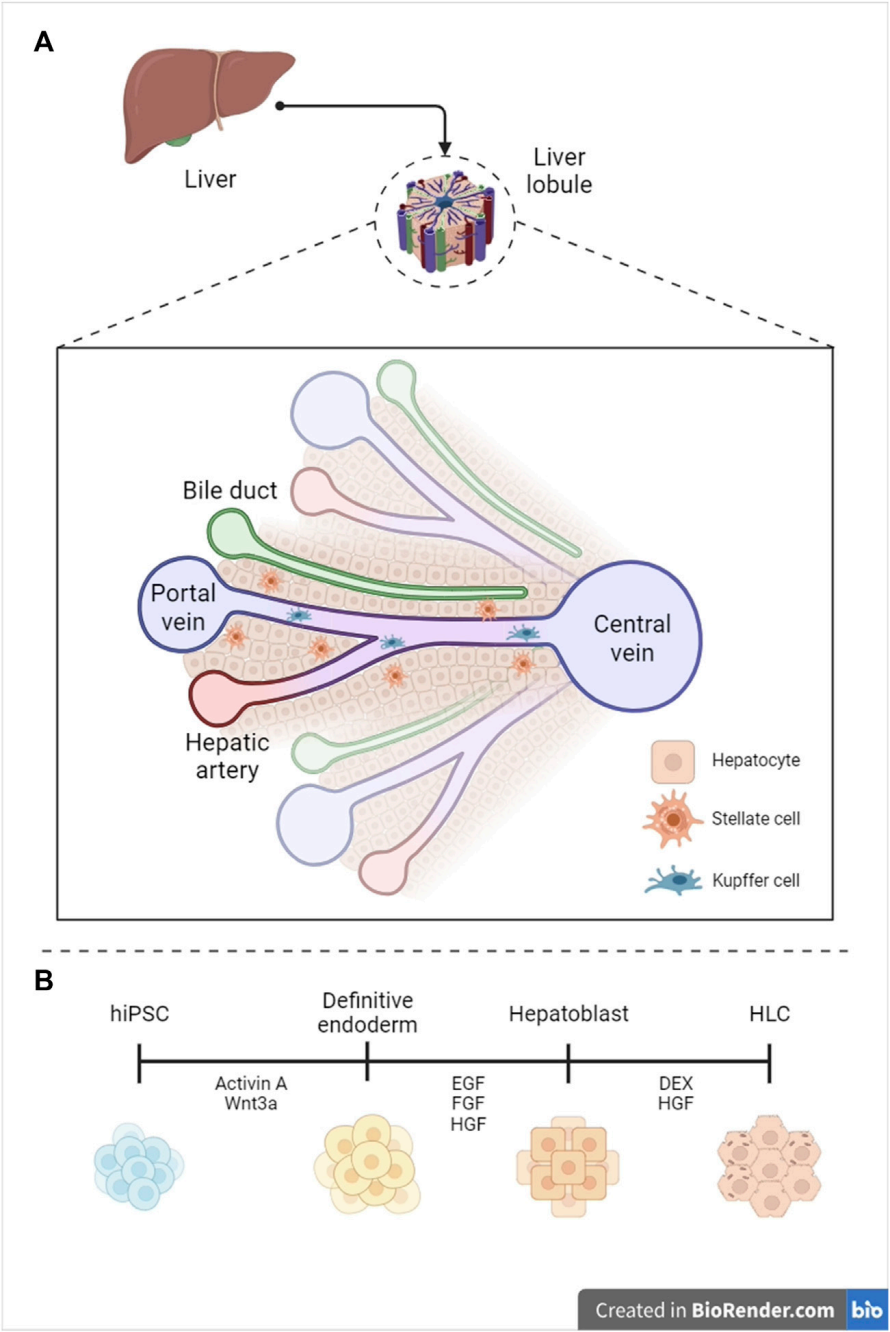
Graphical representation of liver anatomy **(A)** and hepatocyte differentiation standard protocol **(B)**. hPSC = human pluripotent stem cell, HLC = hepatocyte-like cell (Created with BioRender.com, modified from (Telles-Silva et al. (2022)), with permission from the authors).

The liver is the second largest organ in humans–constituting 3% of the total body mass–and the most vascularized one, receiving 25% of the cardiac output. Vascularization correlates with liver’s endocrine, exocrine, and metabolic functions, encompassing regulation of blood homeostasis, bile synthesis, detoxification of xenobiotics, and glycogen storage (Wang et al., 2021). The liver can be subdivided into hexagonal lobules, each containing many ascini–the fundamental hepatic functional units. The acinus is a cross-section between two adjacent portal triads, comprising the portal vein, hepatic artery, and intrahepatic bile ducts (zone 1, or periportal), and two adjacent central veins (zone 3, pericentral) (Figure 1A) (Abdel-Misih and Bloomston, 2010). Parenchymal hepatocytes populate the liver plates and account for the core hepatic metabolism, while non-parenchymal cells, including stellate, Kupffer and liver sinusoidal endothelial cells, play an essential role in extracellular matrix composition (Kamm and McCommis, 2022), immune response regulation (Li et al., 2022), and vascular tone modulation (Gracia-Sancho et al., 2021), respectively.

Liver diseases are a global concern, contributing to two million deaths annually (Ehrlich et al., 2019). These conditions encompass, according to the new nomenclature (Rinella et al., 2023): alcohol-associated liver disease (ALD) (Moradi et al., 2020), metabolic dysfunction-associated steatotic liver disease (MASLD), which includes metabolic dysfunction-associated steatohepatitis (MASH) (Soret et al., 2021), as well as Hepatitis B and C (Kulkeaw and Pengsart, 2021), and drug-induced liver injury (DILI) (Skardal et al., 2020). If left untreated, liver diseases progress to severe prognostic conditions, such as cirrhosis and hepatocellular carcinoma (HCC), often culminating in irreversible liver failure (Younossi et al., 2019).

End-stage liver disease is currently managed solely through transplantation. In 2023, the transplant waiting list saw the addition of 122 patients each day, marking a 6.9-fold increase compared to 2022 (Organ Procurement and Transplantation Network, 2022). Given the poor prognosis associated with end-stage liver failure and the significant financial burden it imposes on the healthcare system, any advancement capable of reducing transplantation waitlists would not only extend patient lifespans but also optimize healthcare expenditures (Hannezo and Heisenberg, 2019). However, it is crucial to note that artificial liver replacement remains an unmet need, and the shortage of liver grafts remains a pressing global issue.

The dependence on limited donors has required the development of alternative treatments based on bioengineering. Recellularization and bioprinting are two promising approaches to end organ shortage (Hunsberger et al., 2016). Scaffolds can be generated through cellular removal (i.e., decellularization) or by additive manufacturing using biomaterials to replicate the liver’s microenvironment. Both decellularized and bioprinted scaffolds need to be populated with millions of highly functional allogeneic or autologous liver cells, allowing the recovery of key hepatic functions. Therefore, in this review, we summarize recent differentiation protocols for generating distinct liver cell types and their applications for populating decellularized and bioprinted scaffolds.

## Cell source and hepatocyte differentiation

To generate functional bioprinted and recellularized organs, a reliable cell source is imperative. Primary human hepatocytes (PHHs) represent the gold standard for hepatic research, exhibiting phase I and II metabolism, ammonia detoxification, and glucose synthesis (Desai et al., 2017). Nonetheless, the applicability of PHHs is constrained by their limited availability, low *in vitro* proliferation, and substantial donor-to-donor variability (Lauschke et al., 2016). Immortalized cell lines are the predominant *in vitro* model for hepatic research, presenting low cost and high proliferation. However, HepG2s are cancer-derived and fail to replicate the enzymatic activity of PHHs (Yang et al., 2023). A cell source alternative lies in human induced pluripotent stem cells (hiPSCs), which are reliable due to their sustained proliferation through self-renewal and potential to differentiate into cell lineages stemming from all three germ layers (Yamanaka, 2020). To differentiate hiPSCs into HLCs, researchers replicate the key molecular events that occur during liver embryonic development, subdivided into three consecutive stages: 1) commitment into foregut endoderm, 2) specification into hepatic progenitors, and 3) maturation to HLCs (Figure 1B). We will primarily focus on the last step, which represents a significant barrier to improving hepatocyte functionality.

In 2D *in vitro* hepatic differentiation, the factors HGF and DEX are essential. Although HLCs exhibit albumin synthesis, glycogen storage, indocyanine green (ICG) uptake, low-density lipoprotein (LDL) uptake, and urea secretion, they also express fetal liver markers (e.g., *AFP*, *FOXA2* and *GATA6*) and have low CYP450 activity, akin to hepatocytes from <20 weeks of gestation (Funakoshi et al., 2011). Therefore, achieving terminal hepatic differentiation *in vitro* remains a challenge (Raggi et al., 2022).

To address the limited functionality of 2D HLCs, researchers have enriched differentiation media, incorporated cell-cell and cell-extracellular matrix interactions, and adopted multilayer and multicellular approaches (Mun et al., 2019). Ardisasmita et al. (2022) conducted a robust transcriptomic analysis of a myriad of HLCs from multiple studies, revealing a high transcriptome similarity between HLCs and PHHs (Ardisasmita et al., 2022). However, the proteome maturation, including abundance, distribution, post-translational modifications, and activity, is crucial for hepatocyte functionality and does not strictly correlate with transcriptome observations (Hurrell et al., 2019). Altogether, modifying the differentiation media composition improves the differentiation efficiency, but 2D models reach a plateau that is only trespassable by increasing the complexity of the cell culture model to better reproduce the liver microenvironment. Therefore, the growing adoption of 3D culture models, which mimic *in vivo* cell interactions during the embryonic development, has led to the production of more mature cells.

Organoids are the most used 3D platforms for liver differentiation. These mini-livers consist of parenchymal hepatocytes and cholangiocytes, as well as non-parenchymal cell types like Kupffer, stellate, and sinusoidal endothelial cells, all of which are critical for maintaining homeostasis and responding to the progression of liver disease (Koike et al., 2019). Ouchi et al. (2019) co-differentiated epithelial and stromal lineages from PSCs in a novel organoid culture, replicating the presence of supportive lineages on the liver, such as hepatic stellate cells and Kupffer cells (Ouchi et al., 2019). However, we note that organoids are heterogeneous, frequently irreproducible between batches, and necrotic at their core, reproducing liver function better than other *in vitro* models, but lacking microstructure accuracy (Harrison et al., 2021).

## Application of hiPSCs in liver tissue engineering

Orthotopic liver transplantation currently stands as the sole efficacious treatment for severe hepatic failure. However, the scarcity of suitable donors for transplantation renders this solution impracticable. In response, researchers are increasingly turning to bioengineering models to alleviate the demand for donors in liver transplantation, especially recellularization of decellularized and bioprinted liver scaffolds (Reza et al., 2021).

## hiPSC-derived hepatocytes for recellularization

Decellularization was one of the pioneering scaffold-based approach to replace the need for organ donors (Messina et al., 2020). Decellularization encompasses liver perfusion or full immersion in detergents (e.g., SDS, Triton X-100), which lyse cells without disrupting the tissue microstructure, followed by multiple saline solution washes (Ahmed et al., 2019). Enzymatic (e.g., nuclease, trypsin) or mechanical (e.g., agitation, pressurization, and shear stress) decellularization can be employed alone or in conjunction with detergents to enhance the removal of cell lysis residues (Faccioli et al., 2020).

After decellularization, the extracellular matrix graft (ECM-G) can be repopulated with organ specific cell types that adhere to the tissue microstructure, constituting the recellularization. Two key factors determine recellularization efficiency: 1) the method of cell deposition/reinsertion, and 2) the cell type. Static cell deposition involves injecting concentrated cell suspensions into the scaffold, resulting in limited cell penetration, seeding, and reduced microvessel establishment, yielding an overall efficacy ranging from 10%–25% (Watanabe et al., 2019). Active deposition, on the other hand, entails cell perfusion via the liver native vascular network, often via cannulation of the portal and central veins, leading to seeding efficacy as high as 85% (Asadi et al., 2021).

Various cell types, including PHHs, immortalized cell lines, and stem cell-derived cells, have been employed in the recellularization of human and non-human scaffolds (Antarianto et al., 2022). Acun et al. (2022) recellularized rat liver scaffolds with iPSCs and differentiated them into HLCs by perfusing defined factors. The resulting HLCs exhibited high expression of mature hepatic markers (e.g., *HNF4A*, *CYP3A4*, *CEBPA* and *ALB*), low expression of *AFP,* and a progressive increase in urea synthesis and albumin secretion (Acun et al., 2022). However, successful recellularization is not restricted to liver scaffolds. Abazari et al. (2020) generated HLCs with stable albumin secretion, urea synthesis, and Cyp3a4 activitywithin a decellularized amniotic membrane, underscoring the potential of non liver-specific ECM-G for deriving functional cells (Abazari et al., 2020).

To produce liver grafts compatible with transplantation, researchers are increasingly implementing hiPSC-derived hepatic cell lines for recellularization. Minami et al. (2019) recellularized a rat ECM-G with HLCs expressing *ALB* and *CYP3A4* after 48 h of perfusion (Minami et al., 2019). However, elevated *AFP* expression and reduced albumin secretion indicated an immature phenotype in comparison to recellularization with PHHs. To evaluate the functionality of ECM-Gs within a physiological context, Kojima et al. (2022) transplanted a scaffold recellularized with human HLCs into a pig model. The graft retained liver markers and viability for 28 days, after which the absence of microvasculature induced cell death (Kojima et al., 2022).

Although decellularization preserves vascular microstructures, anastomosis alone is insufficient to maintain hemocompatibility. To address thrombus formation, researchers are enhancing endothelization during recellularization through gene editing. Hu et al. (2020) achieved increased vascular bed formation by overexpressing Syndecan-4 in endothelial cells, leading to competitive inhibition of THBS1 (Hu et al., 2020). However, overexpressing Syndecan-4 is associated with carcinogenesis (Habes et al., 2019). Therefore, further studies are warranted to assess the efficacy and the safety of editing distinct gene subsets.

We conclude, therefore, that liver decellularization has been demonstrating clear progress in efficiency (Rossi et al., 2019), although recellularization needs further improvement for suiting transplantation, especially regarding cell coverage (usually <75%), cell diversity, functionality, and long-term maintenance (Dai et al., 2022).

## hiPSC-derived hepatocytes for bioprinting

Biofabrication, defined as the automated production of functional tissues and organs, is a burgeoning field (Ambhorkar et al., 2020). Within the area, three-dimensional (3D) bioprinting holds promise for the manufacturing of artificial organs, enabling not only precise cell deposition, but shape and size control combining biocompatible bioinks and bioprinting techniques (He et al., 2022).

Bioinks can be generated from natural components (e.g., decellularized liver ECM, collagen, gelatin, alginate) or synthetic polymers (e.g., polyethylene glycol, polycaprolactone, polyvinylpyrrolidone) (Khoeini et al., 2021). Blackford et al. (2023) reported that HLCs cultured on soft polyethylene glycol (2.5%) secreted more ALB than PHHs and metabolized more urea than HLCs cultured in alginate (Blackford et al., 2023), underscoring the potential of synthetic polymers for bioprinting. Additionally, Abbasalizadeh et al. (2023) achieved a high yield of hiPSC-derived hepatic organoids by combining PEG microencapsulation and microfluidic systems, paving the way for cell transplantation (Abbasalizadeh et al., 2023).

In parallel to the bioink source, researchers produce hepatic microtissues through three primary printing methods: 1) extrusion-based, 2) stereolithography-based, or 3) laser-assisted.

Extrusion-based techniques entail bioprinting viscoelastic bioinks, with or without a cell suspension. Given the wide variety of commercially available extrusion-based bioprinters, this method is cost-effective and reliable (Khoeini et al., 2021). Goulart et al. (2019) successfully bioprinted iPSC-derived liver spheroids using bioink extrusion of an alginate/pluronic blend (Goulart et al., 2019). This resulted in increased urea synthesis, extended albumin secretion, and elevated gene expression of phase one metabolism enzymes. Despite the widespread use of extrusion-based scaffolds, they often require bioink crosslinking, which can lead to suboptimal long-term stability, and have low cell density and limited resolution.

Stereolithography utilizes light-dependent polymerization, resulting in accelerated bioprinting and enhanced scaffold stability (Khoeini et al., 2021). Sphabmixay et al. (2021) crafted elastic hepatic lobules that mimicked the native liver parenchymal and vascular microstructure, all while maintaining cell function for 7 days (Sphabmixay et al., 2021). Similarly, Wesseler et al. (2023) developed liver microphysiological systems that replicated physiological oxygen gradients and sustained the growth of HLCs. However, it is important to note that the technical expansion of stereolithography faces challenges, primarily stemming from the limited diversity of photosensitive bioinks and potential cell toxicity during UV-based polymerization.

Laser-assisted fabrication employs a laser beam to transfer energy to a metal-absorbing layer, leading to the precipitation of a cell-blended bioink from a coating layer into a reservoir created through beam scanning or image projection. This method allows for bioink deposition without the need for a nozzle, resulting in high-resolution, stable, and densely populated bioprinted structures (Khoeini et al., 2021). Koch et al. (2018) pioneered the laser printing of HLCs, underscoring the relevance of suitable hydrogels and sols for cell maintenance and function (Koch et al., 2018). While laser constructs exhibit functionality, their high cost limits extensive use in basic research (Ventura, 2021). However, co-culturing HLCs with endothelial cells can enhance the applicability of laser bioprints for disease modeling, potentially justifying the budget for applied research.

3D bioprinting provides an effective strategy for replicating the native liver microenvironment at both structural and functional levels, allowing for the recreation of liver lobule microstructures and enhancing the metabolism of HLCs (Agarwal et al., 2021). To develop constructs compatible with transplantation, it is crucial to reproduce the intrahepatic microstructure (Wang et al., 2022). Fukumitsu et al. (2023) printed hepatic models based on computed tomography liver imaging to facilitate pre-operative plans. This approach paves the way for generating personalized bioprinted livers not only by repopulating the constructs with autologous iPSC-derived cells but also by mimicking the patient’s vascular and biliary networks. However, the authors used a synthetic ink for the liver model, which is incompatible with the goals of personalized medicine, emphasizing the need of further research into anatomically functional stable, and biocompatible constructs.

Nevertheless, given the early stage of this technology in tissue engineering, addressing the challenges of vascularization, reproducibility, and scalability requires further model optimization on both the biomaterial (i.e., bioink composition, stiffness, and thickness) and cell (i.e., co-culture, functionality, and vascularization) fronts (Ma et al., 2020).

## Discussion

In the realm of cell manufacturing and sourcing, hiPSCs are catalyzing a revolution in disease modeling, therapy development, and personalized medicine (Napolitano et al., 2021). The capacity to differentiate hPSCs into every single human tissue, facilitated by growth factors, small molecules, and culture plates coated with ECM-like solutions (e.g., Matrigel, Geltrex), enables the *in vitro* mimicking of organ development.

Summarizing recent liver differentiation protocols, we evidenced the challenges in achieving the phenotype of native cells *in vitro* that persist across distinct tissues. Firstly, the use of complex supplements (e.g., KOSR) and coating solutions (e.g., Matrigel and Geltrex) simplifies cell culture routines and reduces experimental costs. However, these may compromise the precise tuning of cell fate determination–while complex supplements exhibit batch-to-batch variation (Bushweller et al., 2022) -, and animal-derived reagents reduce the efficiency of hPSCs differentiation (Saari et al., 2022). Secondly, classical monolayer (2D) cell culture and, consequently, hPSC differentiation inadequately mimic embryonic and tissue development, as gene regulation and morphogenesis critically depend on cell-cell and cell-ECM three-dimensional interactions (Jensen and Teng, 2020). Finally, reprogrammed hiPSCs retain epigenetic signatures of donor cells, limiting their efficiency in differentiating into alternative lineages (Calabrese et al., 2019).

Despite the challenges in hPSC differentiation protocols and applications, the strategies for overcoming them are aligned. Formulating chemically defined differentiation media employing animal origin-free (AOF) matrix components (e.g., fibronectin, laminin, poly-L-ornithine) standardizes cellular phenotypes (Pan et al., 2021). 3D-cell differentiation methods (spheroids, organoids, bioprinting, and bioreactors) enable the spontaneous or designed reconstruction of the developing tissue microenvironment (Xie et al., 2021). However, a remaining challenge for the therapeutic implementation of iPSC-derived liver cells is safety. iPSCs not infrequently exhibit concerning genomic and epigenomic aberrations (e.g., aneuploidies, translocations), which are retained by differentiated cells and may elevate the risk of tumorigenicity during *in vivo* long-term expansion (Zhang et al., 2021). Therefore, additional research with animal models is imperative to investigate the outcomes of transplanting scaffolds and bioprinted constructs recellularized with iPSC-derived cells.

Immunosuppression has been instrumental in graft survival and prevention of rejection following organ transplantation (Hunsberger et al., 2016). However, long-term immunosuppression carries several side-effects, including reduced quality of life, potential damage to the transplanted organ if not managed, and an increased risk of infection. The use of autologous cells can mitigate rejection and reduce the need for immunosuppression. Nevertheless, allogeneic sources are more cost-effective, with gene editing shedding light to the generation of universal cell lines for bioengineering applications. Complementarily, decellularized scaffolds align with hiPSC-derived cells to prevent organ rejection since no significant DNA and proteins remain after cell removal (Caires-Júnior et al., 2021).

Vascularization and innervation are not reestablished at the time of organ transplant (Hunsberger et al., 2016). Recellularizing biological scaffolds and bioprinted structures with hiPSC-derived liver-specific endothelial cells (Gage et al., 2020) or endothelial-containing liver organoids (Pettinato et al., 2019) enhances tissue integration within transplantation. Furthermore, inducing and regulating long-term vascularization remains a challenge. To enhance the establishment of a vascular network, small molecules and proteins induce endothelial cell proliferation and growth. However, stimulating cell proliferation *in vivo* is not cell type specific and represents a significant barrier, as the risk of carcinogenicity is proportionally increased (Mir et al., 2023). Consequently, researching the combination of distinct tissue-specific cell types and the vascularization by gene editing aligns with bioengineering goals, improving transplantation efficiency, organ functionality, and transplant lifespan (Yang et al., 2021).

Additional challenges encompass the storage and commercialization of artificial organs. Recent breakthrough research in cryobiology, including liver subzero preservation techniques (Ozgur et al., 2023), allow for incremental organ transportation by preserving the structure of decellularized organs or minimizing cell damage for recellularized ones. Ensuring construct stability is a significant challenge in translating bioprinting for transplantation. Synthetic biomaterials (e.g., PCL, PEG, PLGA) yield more stable structures but may elicit immunogenic responses. Conversely, while natural bioinks (e.g., alginate, collagen, gelatin) are biocompatible and degradable, achieving optimal printability requires precise adjustment of bioprinting parameters (e.g., bioink concentration, printing time, crosslinking strategy) and often involves blending with other biomaterials (Fang et al., 2023).

Despite remaining difficulties in translating *in vitro* advances with liver modeling to clinical trials, recent strides in bioengineering with hiPSC-derived cells point toward the establishment of tangible alternatives to traditional donor-dependent organ transplantation. Thus, we posit that the combination of iPSC-derived cells with decellularized matrices and bioprinted constructs will facilitate technological translation towards personalized medicine, creating alternative organ sources for liver transplantation.
